# Responses of five small mammal species to micro-scale variations in vegetation structure in secondary Atlantic Forest remnants, Brazil

**DOI:** 10.1186/1472-6785-8-9

**Published:** 2008-05-05

**Authors:** Thomas Püttker, Renata Pardini, Yvonne Meyer-Lucht, Simone Sommer

**Affiliations:** 1 Leibniz-Institute for Zoo- and Wildlife Research, Alfred-Kowalke-Str. 17, D-10315 Berlin, Germany; 2 Universidade de São Paulo, Instituto de Biociências, Rua do Matão, travessa 14, 101/Departamento de Zoologia, Cidade Universitária, 05508-900, Sao Paulo, Brasil

## Abstract

**Background:**

The Brazilian Atlantic Forest is highly endangered and only about 7% of the original forest remains, most of which consists of fragments of secondary forest. Small mammals in the Atlantic Forest have differential responses to this process of fragmentation and conversion of forest into anthropogenic habitats, and have varying abilities to occupy the surrounding altered habitats. We investigated the influence of vegetation structure on the micro-scale distribution of five small mammal species in six secondary forest remnants in a landscape of fragmented Atlantic Forest. We tested whether the occurrence of small mammal species is influenced by vegetation structure, aiming to ascertain whether species with different degrees of vulnerability to forest fragmentation (not vulnerable: *A. montensis*, *O. nigripes *and *G. microtarsus*; vulnerable: *M. incanus *and *D. sublineatus*; classification of vulnerability was based on the results of previous studies) are associated with distinct vegetation characteristics.

**Results:**

Although vegetation structure differed among fragments, micro-scale distribution of most of the species was influenced by vegetation structure in a similar way in different fragments. Among the three species that were previously shown not to be vulnerable to forest fragmentation, *A. montensis *and *G. microtarsus *were present at locations with an open canopy and the occurrence of *O. nigripes *was associated to a low canopy and a dense understory. On the other hand, from the two species that were shown to be vulnerable to fragmentation, *M. incanus *was captured most often at locations with a closed canopy while the distribution of *D. sublineatus *was not clearly influenced by micro-scale variation in vegetation structure.

**Conclusion:**

Results indicate the importance of micro-scale variation in vegetation structure for the distribution of small mammal species in secondary forest fragments. Species that are not vulnerable to fragmentation occurred at locations with vegetation characteristics of more disturbed forest, while one of the species vulnerable to fragmentation was found at locations with older forest characteristics. Results suggest that micro-habitat preferences may be an important factor influencing the capacity of small mammals to occupy altered habitats and, consequently, their vulnerability to forest fragmentation at a larger spatial scale.

## Background

The coastal Atlantic Forest is one of the most diverse, and most threatened, natural environments in the world [1]. Due to severe human impact over the last few centuries most of the primary coastal Atlantic Forest has been destroyed. Only 7% of its original extent remains [1] and a considerable part of remaining forest takes the form of secondary forest fragments at different stages of regeneration, embedded in a rural or urban matrix [2,3]. In fragmented landscapes such as these, both the stage of regeneration and the degree of fragmentation influence forest structure within the fragments, which in turn determines habitat suitability and affects species occurrence as well as the composition of animal communities [4,5]. Since secondary forests may play an important role in species conservation if primary forest habitats are limited [5], it is important to understand which factors determine species distribution in secondary forests.

Although small mammals play a key role in neotropical forest ecosystems, including the Atlantic Forest, in terms of seed dispersal [6-11], dispersal of mycorrhizal fungi [12] and predation on seedlings [7,10,13,14], only a limited number of studies focused on the influence of vegetation structure on small mammal distribution in secondary Atlantic Forest fragments [15-17].

The effects of forest fragmentation on Atlantic Forest small mammals vary among species. Some species are able to persist in isolated fragments and cross or occupy non-native vegetation in human-altered areas, while others are restricted to large and/or connected forest remnants [16,18-22]. Since vulnerability to fragmentation is influenced by the capacity to occupy the altered habitats of the matrix [19,21,23-25], it is expected that preferences on a microhabitat scale in forested habitats will vary among species showing different degrees of vulnerability to forest fragmentation on a larger scale.

The identification of the scale that best explains variation in presence or abundance of organisms is a major goal in ecology [26]. This implies the necessity of examining ecological patterns on different scales. The issue of the "correct" scale at which assessment of biological parameters should be carried out has been the topic of numerous studies in ecology [27-32]. In fact, in his review Jorgensen [32] called attention to the lack of consistent use of the appropriate scale in small mammal microhabitat research and that broad but widely accepted generalizations (e.g. habitat partitioning, relation between abundance and availability of microhabitat) might be based on results that often confound micro- and macro-habitat effects.

In a recent study on small mammals in Caucaia do Alto, São Paulo, Pardini et al. [16] showed that three rodent and two marsupial species were affected in different ways by fragmentation on a macro-scale. The rodents *Akodon montensis *Thomas, 1902, and *Oligoryzomys nigripes *(Olfers, 1818) as well as the marsupial *Gracilinanus microtarsus *(Wagner, 1842) were not affected by fragmentation while the rodent *Delomys sublineatus *(Thomas, 1903) and the marsupial *Marmosops incanus *(Lund, 1840) decreased in abundance in smaller and/or more isolated fragments. In the same region, Umetsu & Pardini [21] showed that the three species not affected by fragmentation were able to occupy anthropogenic habitats while the species vulnerable to fragmentation were restricted to native vegetation on a macro-habitat scale [16,21].

In the present study we extended the previous work by adding a smaller scale to the investigation of small mammal habitat preferences in the same Atlantic Forest study area. While Umetsu and Pardini [21] studied the response of Atlantic Forest small mammal populations to habitat variation, this study deals with the response of small mammal individuals to micro-habitat differences. Specifically, we investigated the influence of vegetation structure on the micro-scale distribution of three small mammal species not affected by fragmentation (*Akodon montensis*, *Oligoryzomys nigripes*, *Gracilinanus microtarsus*) and two species vulnerable to fragmentation (*Delomys sublineatus*, *Marmosops incanus*) in six secondary forest remnants in the Atlantic Forest fragmented landscape of Caucaia do Alto. We investigated if the occurrence of small mammal species is influenced by vegetation structure, aiming to ascertain whether species with different degrees of vulnerability to forest fragmentation are associated with distinct vegetation characteristics.

## Results

### Species captured

In total, 698 individuals belonging to 12 species were captured 1597 times in 14400 trap nights, resulting in a trap success of 11.1%. The five focus species were the most common species in the studied sites: the terrestrial rodents *Akodon montensis *and *Delomys sublineatus*, the scansorial rodent *Oligoryzomys nigripes*, the scansorial marsupial *Marmosops incanus *and the arboreal marsupial *Gracilinanus microtarsus *(Table 1), which accounted for 76.8% of individuals captured.

**Table 1:** Investigated species.

Species	Locomotion habits	Number of individuals captured	Trap locations used	Percentage of trap locations with more than one individual captured
*Akodon montensis*	*terrestrial*	284 (140/138/6)	230	34.35
*Oligoryzomys nigripes*	*scansorial*	46 (33/13)	38	15.79
*Delomys sublineatus*	*terrestrial*	83 (37/43/3)	81	16.05
*Marmosops incanus*	*scansorial*	64 (33/31)	72	9.72
*Gracilinanus microtarsus*	*arboreal*	59 (25/33/1)	58	13.79

Locomotion habits ([58, 59] and own observations) of the investigated species, number of individuals captured (numbers of males, females and unsexed individuals are given in parenthesis), number of used trap locations and percentage of trap locations where more than one individual of the same species was captured.

Additional species captured were the sigmodontine rodents *Juliomys spp*. (Osgood, 1933, 32 individuals), *Euryoryzomys russatus *(Wagner, 1848, 20), *Sooretamys angouya *(Fischer, 1814, 26), *Brucepattersonius soricinus *(Thomas, 1896, 5), *Thaptomys nigrita *(Lichtenstein, 1829, 58) and the didelphid marsupials *Didelphis aurita *(Wied-Neuwied, 1826, 23) and *Monodelphis americana *(Müller, 1776, 14), and *Micoureus paraguayanu*s (Tate, 1931, 1).

In several trap locations more than one individual of the same species was captured (Table 1). In the case of *Akodon montensis*, the percentage of traps with more than one individual of the same species was 34.35% (Table 1). For all other species, however, the percentage of traps with more then one individual of the same species was below 20% (Table 1).

### Principal component analysis to identify major traits of variation in vegetation structure

The vegetation characteristics were reduced to three principal components (PC1, PC2, PC3) with an Eigenvalue greater than one, which explained 69.9% of the variance in the data (Table 2). The factor loadings are a measure of the correlation between original habitat variables and the new variables (PC's, [33]). The correlation matrix (Table 2) revealed that the first principal component, which explained 33.9% of the variance, described the canopy height and the density of the vegetation up to three meters. The second principal component (20.5% variance explained) reflected the amount of bamboo and the number of horizontal structures while the third (15.5% variance explained) described the density of the canopy (Table 2).

**Table 2:** Factor loadings of Principal Components.

	PC1	PC2	PC3
Eigenvalue	2.37	1.44	1.09
Variance explained	33.88	20.51	15.50
cumulative Variance	33.88	54.39	69.89

Canopy cover	-0.11	-0.09	0.96
Canopy height	-0.69	0.02	0.24
Vegetation density 0 – 0.5 m	0.80	-0.23	-0.05
Vegetation density 0.5 – 1.5 m	0.87	0.12	-0.03
Vegetation density 1.5 – 3 m	0.69	0.42	0.17
Amount of Bamboo	0.03	0.75	0.08
Amount of horizontal structures	-0.02	0.78	-0.28

Eigenvalues, variance explained and correlations (factor loadings) between the three principal components and vegetation characteristics.

### Species response to micro-scale variation in vegetation structure

The vegetation structure synthesized by all principal components differed among fragments (two-factorial ANOVA; Table 3). Species response to these major trends of vegetation structure variation, however, was in most cases independent of the fragment considered.

**Table 3:** Full results of 2-way-ANOVA. Results of 2-way-ANOVA for all species and all three principal components.

		SS	DoF	MS	F	*p*
***Oligoryzomys nigripes***	**PC1**					
	Intercept	34.19	1	34.19	32.26	< 0.0001
	Fragment	10.67	3	3.56	3.36	0.019
	Use	8.57	1	8.57	8.09	0.005
	Fragment*Use	4.58	3	1.53	1.44	0.230
	Error	424.00	400	1.06		
						
	**PC2**					
	Intercept	0.00	1	0.00	0.00	0,970
	Fragment	13.60	3	4.53	3.71	0.012
	Use	1.53	1	1.53	1.26	0.263
	Fragment*Use	7.89	3	2.63	2.15	0.093
	Error	488.47	400	1.22		
						
	**PC3**					
	Intercept	1.02	1	1.02	1.26	0.262
	Fragment	18.81	3	6.27	7.78	< 0.0001
	Use	1.24	1	1.24	1.54	0.215
	Fragment*Use	2.58	3	0.86	1.07	0.364
	Error	322.46	400	0.81		
						
***Gracilinanus microtarsus***	**PC1**					
	Intercept	4.53	1	4.53	4.72	0.030
	Fragment	27.51	4	6.88	7.16	< 0.0001
	Use	0.00	1	0.00	0.00	0.962
	Fragment*Use	5.28	4	1.32	1.37	0.242
	Error	481.41	501	0.96		
						
	**PC2**					
	Intercept	0.26	1	0.26	0.25	0.618
	Fragment	20.80	4	5.20	4.98	0.001
	Use	0.85	1	0.85	0.82	0.367
	Fragment*Use	6.49	4	1.62	1.55	0.186
	Error	523.52	501	1.04		
						
	**PC3**					
	Intercept	2.46	1	2.46	2.97	0.085
	Fragment	68.78	4	17.19	20.78	< 0.0001
	Use	9.54	1	9.54	11.53	0.001
	Fragment*Use	2.97	4	0.74	0.90	0.465
	Error	414.46	501	0.83		
						
***Akodon montensis***	**PC1**					
	Intercept	1.79	1	1.79	2.19	0.140
	Fragment	75.06	5	15.01	18.29	< 0.0001
	Use	49.27	1	49.27	60.05	< 0.0001
	Fragment*Use	40.99	5	8.20	9.99	< 0.0001
	Error	586.73	715	0.82		
						
	**PC2**					
	Intercept	0.01	1	0.01	0.02	0.900
	Fragment	44.68	5	8.94	9.44	< 0.0001
	Use	6.83	1	6.83	7.21	0.007
	Fragment*Use	12.28	5	2.46	2.59	0.025
	Error	677.11	715	0.95		
						
	**PC3**					
	Intercept	1.82	1	1.82	2.25	0.134
	Fragment	103.50	5	20.70	25.67	< 0.0001
	Use	4.81	1	4.81	5.97	0.015
	Fragment*Use	5.07	5	1.01	1.26	0.281
	Error	576.,55	715	0.81		
						
***Delomys sublineatus***	**PC1**					
	Intercept	2.87	1	2.87	4.53	0.034
	Fragment	36.25	4	9.06	14.32	< 0.0001
	Use	0.02	1	0.02	0.03	0.854
	Fragment*Use	1.81	4	0.45	0.72	0.581
	Error	318.98	504	0.63		
						
	**PC2**					
	Intercept	0.50	1	0.50	0.49	0.482
	Fragment	30.53	4	7.63	7.58	< 0.0001
	Use	1.40	1	1.40	1.40	0.238
	Fragment*Use	5.37	4	1.34	1.33	0.256
	Error	507.31	504	1.01		
						
	**PC3**					
	Intercept	2.05	1	2.05	2.47	0.117
	Fragment	51.46	4	12.87	15.50	< 0.0001
	Use	1.91	1	1.91	2.30	0.130
	Fragment*Use	1.79	4	0.45	0.54	0.706
	Error	418.38	504	0.83		
						
***Marmosops incanus***	**PC1**					
	Intercept	2.08	1	2.08	2.47	0.117
	Fragment	38.28	5	7.66	9.10	< 0.0001
	Use	3.65	1	3.65	4.34	0.038
	Fragment*Use	3.07	5	0.61	0.73	0.601
	Error	500.42	595	0.84		
						
	**PC2**					
	Intercept	2.94	1	2.94	3.22	0.073
	Fragment	39.16	5	7.83	8.57	< 0.0001
	Use	3.98	1	3.98	4.36	0.037
	Fragment*Use	8.75	5	1.75	1.92	0.090
	Error	543.80	595	0.91		
						
	**PC3**					
	Intercept	1.65	1	1.65	1.95	0.163
	Fragment	58.41	5	11.68	13.82	< 0.0001
	Use	2.51	1	2.51	2.97	0.085
	Fragment*Use	2.44	5	0.49	0.58	0.718
	Error	503.02	595	0.85		

#### Species non-vulnerable to fragmentation

**Figure 1:**
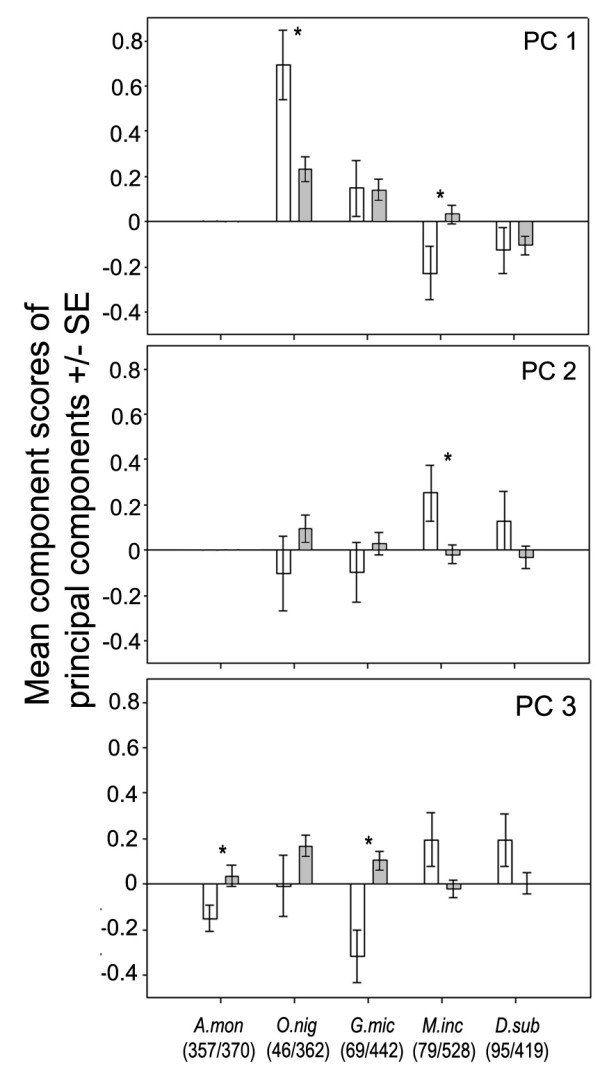
**Association between the occurrence of small mammal species and vegetation characteristics described by the three principal components**. Comparison of mean (+/- standard error) component scores of used (open bars) and unused (grey bars) trap locations for *A. montensis *(A. mon), *O. nigripes *(O. nig), *G. microtarsus *(G. mic), *D. sublineatus *(D. sub), and *M. incanus *(M. inc) and. Asterisks mark a significant difference between used and unused trap locations. Numbers of used/unused trap locations are given below. For *A. montensis *results of PC1 and PC2 are not given because the use of vegetation described by these variables differed between fragments (significant interaction between FRAGMENT and USE).

*O. nigripes *was captured at trap locations with a significant higher mean value for PC1 (Table 3; Fig. 1), meaning a lower canopy and denser understory vegetation. No significant difference was found between used and unused trap locations considering PC2 or PC3 (Table 3; Fig. 1), as well as, no significant interaction between factors (FRAGMENT*USE; Table 3).

The marsupial *G. microtarsus *was captured at locations with a lower mean value for PC3 (Table 3; Fig. 1) i.e. locations with lower canopy cover. Mean values between used and unused trap locations did not differ significantly in PC1 or PC2 (Table 3; Fig. 1) and again no significant interaction between factors (FRAGMENT*USE) was found (Table 3).

For *A. montensis*, there was a significant difference between used and unused trap locations in all principal components (Table 3). Significant interactions between factors FRAGMENT*USE, however, was observed for PC1 and PC2 (Table 3), indicating that the influence of vegetation structure synthesized in these two principal components on the species distribution was depended on the fragment considered. Irrespective of the fragment, however, *A. montensis *similarly to *G. microtarsus *was captured in trap locations with a significantly lower mean value for PC3, i.e. lower canopy cover (Fig. 1).

#### Species vulnerable to fragmentation

For *D. sublineatus*, mean values did not differ significantly between used and unused trap locations (Table 3; Fig. 1) as well as no significant interaction between factors FRAGMENT*USE was found in any of the principal components (Table 3).

For *M. incanus*, however, there was a significant difference between mean values of used and unused trap locations in PC1 and PC2 (Table 3; Fig. 1), meaning that the species was captured more often in locations with higher canopy and less dense understory as well as in locations with a higher amount of horizontal structures. No significant interaction between factors FRAGMENT*USE was found for any of the principal components (Table 3).

## Discussion

This study examined the response of individuals from five small mammal species to vegetation structure on a micro-scale and represents an extension of former studies by Pardini et al. [16] and Umetsu and Pardini [21] which assessed the influence of a larger scale (habitat) variation in vegetation structure and fragment size on small mammal populations in the same study area.

The results presented here revealed differences in vegetation structure among fragments as well as within fragments among trap locations. The observed major micro-scale trends in vegetation structure (as synthesized by the three principal components) within the secondary forest fragments studied represented a gradient of forest succession/disturbance from old/undisturbed sites (higher canopy height, less dense understory, less horizontal structures, denser canopy cover) to initial/disturbed sites (lower canopy height, denser understory, more horizontal structures, less dense canopy cover), similar to that observed on a larger scale (among habitat fragments) in Caucaia, from fragments of native vegetation in initial stage of regeneration (shrub vegetation) to fragments of native forest [16,21].

These micro-scale differences in vegetation structure are probably caused by the interaction of several factors. Several studies pointed out a variety of factors influencing forest succession [34,35]. There are differences in colonization ability among plant species [35] which may lead to small scale differences in species composition and therefore secondary forest structure. Furthermore, abiotic and biotic conditions might differ between locations. For example, soil nutrients provide an important resource for colonizing plant species after forest clearing [35,36] and are likely to vary due to pre-abandonment management. Furthermore, it has been hypothesized that differences in the occurrence of mycorrhizae might influence vegetation composition [37] and thus vegetation structure. Another cause of micro-scale variation in vegetation structure might be sporadic residual old-growth trees within the secondary vegetation [34] which can influence the surrounding habitat and also substantially increase canopy height at certain locations. Tree falls and the resulting gaps represent an additional source of small-scale variation within the forest [38]. Gaps in the canopy alter microclimatic conditions such as radiation, heat fluxes, wind, and humidity levels [39]. Other possible causes of variation are fire or cattle entering the forest. Although these events were not observed in the studied fragments during the study, they were relatively common in adjacent fragments and could have influenced studied fragments prior to data collection. Because not all parts of the trapping grids were located in equal distance from fragment edges, part of the variation might be due to edge effects. Because the proximity to edges influences several abiotic (micro-climate, windspeed, soil nutrients [24,40]) and biotic (invasion of non-forest species, species composition [41]) aspects of the habitat, edge effects may have severe influences on forest structure [42,43].

Although vegetation structure differed among studied fragments most species responses to micro-scale variation were independent of the fragment considered, i.e. were similar in all fragments. The exception was some of the responses of one species non-vulnerable to fragmentation (*A. montensis*), which were influenced by the fragment considered. Thus, irrespective of the variation in vegetation structure among fragments, the distribution of all species except from *D. sublineatus *was influenced by micro-scale variation (within fragment) in vegetation structure. *A. montensis *and *G. microtarsus *were associated to locations with less canopy cover, while *O. nigripes *preferred locations with lower canopy and denser understory. On the contrary, *M. incanus *was captured in locations with higher canopy, less dense understory and higher amount of horizontal structures, while the distribution of *D. sublineatus *was not influenced by within-fragment vegetation variation.

The response group which preferred younger/more disturbed forest sites (*A. montensis*, *O. nigripes*, *G. microtarsus*) included a marsupial and rodents, terrestrial (*A. montensis *[44]) and scansorial (*O. nigripes*, *G. microtarsus *[44-46]) species, as well as insectivores/frugivores (all species [47-49]) and granivores (rodents [10,50]), making taxonomic or ecological traits such as locomotor abilities or diet preferences poor predictors of the observed micro-habitat preferences. Nonetheless, both population response to larger scale (habitat) variation and to fragmentation are consistent with the micro-habitat preferences of this group: species preferring younger/more disturbed sites within secondary forest fragments (*A. montensis*, *O. nigripes *and *G. microtarsus*) were found to be able to occupy anthropogenic habitats and to be equally abundant in smaller and larger forest fragments [16,21].

On the contrary, the marsupial *M. incanus*, which preferred older/less disturbed sites inside secondary forest fragments was found to be restricted to patches of native vegetation and less common in initial stages of regeneration [21]. Although *D. sublineatus *was found to decrease in abundance in smaller and/or isolated forest fragments [16] and to be restricted to patches of native vegetation [21], this species presents similar abundance between patches of native vegetation in initial or latter stages of regeneration (22). Accordingly, we could not find any response of this species to micro-scale vegetation variation inside forest fragments.

Thus, our results indicate that patterns of habitat preference at the population level may be caused by individual responses at a smaller spatial scale (i.e., micro-habitat selection). Species whose individuals prefer younger/more disturbed sites within secondary forest fragments would be able to establish populations in degraded habitats at larger spatial scales, thus being less vulnerable to fragmentation in the long run. On the other hand, species that do not show this preference for disturbed sites or whose individuals are restricted to old/non disturbed sites inside secondary forest fragments would not be able to established populations in degraded open habitats at larger spatial scales, being more vulnerable to fragmentation in the long run.

Studies on the responses of Atlantic Forest small mammals to habitat variation that include different spatial scales are rare (but see [51]). To our knowledge only two studies on microhabitat selection of small mammals in the Atlantic forest biome included partly the same species as in this study [15,17]. Fonseca and Robinson [15] investigated the selection of microhabitat from available habitat (measured as the mean of the variables obtained over all trapping stations) in primary and secondary semi-deciduous Atlantic forest for some small mammal species including *M. incanus *(all other species investigated by Fonseca and Robinson [15] were not subject to this study). They found no habitat selection in *M. incanus*, i.e. this species chose the microhabitat randomly from the available habitat. Dalmagro and Vieira [17] studied microhabitat of *O. nigripes *and *A. montensis *as well as of *Delomys dorsalis*, a congeneric of *D. sublineatus*, in Araucarian forest in south Brazil. Concurring with our results, they found that *O. nigripes *preferred areas in early successional stages within the forest. On the other hand, they also found that *D. dorsalis *preferred denser canopy cover and a positive correlation between canopy cover and the occurrence of *A. montensis*. Although it is difficult to directly compare these results given the heterogeneity in sampling design and data analysis among studies, these results may indicate that the response of the studied species to vegetation structure may vary among different types of forest in the Atlantic forest biome.

## Conclusion

Results showed that major trends in micro-scale variation in vegetation structure within secondary forest fragments are similar to those observed at a larger spatial scale, i.e. among habitat fragments. This micro-scale variation in vegetation structure was shown to influence the distribution of most small mammal species in secondary forest fragments. Species that were shown to be able to establish populations in altered habitat and not to be vulnerable to fragmentation were clearly associated to younger/more disturbed sites within secondary forest remnants. On the contrary, species that were shown to be unable to establish populations in altered habitat and to be vulnerable to fragmentation preferred older/less disturbed sites or were not influenced by micro-scale variation in forest structure inside secondary forest fragments.

Thus the results, in combination with previous studies [16,21], show that species responses are similar at different spatial scales of habitat and microhabitat, suggesting that microhabitat preferences may be an important factor influencing the capacity of small mammal to occupy altered habitats and, consequently, their vulnerability to forest fragmentation at a larger spatial scale.

## Methods

### Study area

The study was conducted in the region of Caucaia do Alto (23°40' S, 47°01' W) about 80 km south-west of the city of São Paulo, in a transition zone between dense ombrophilous forest and semi-deciduous forest classified as "Lower Montane Atlantic Rain Forest" [52] in the State of São Paulo, Brazil. Elevation ranges from 800 to 1100 m [53]. Monthly mean temperature range from a minimum of 11°C to a maximum of 27°C. The annual precipitation level is 1300–1400 mm and fluctuates seasonally with the driest and coldest months between April and August.

The area includes a fragmented landscape, composed of secondary forest fragments embedded in an agricultural landscape, and an adjacent 10.000 ha forest reserve (Morro Grande Forest Reserve). Secondary forest covers 31% of the fragmented landscape, which is dominated by anthropogenic altered habitat (33% agricultural fields, 15% areas with rural buildings or urban areas, 10% vegetation in early stages of regeneration, 7% pine and eucalyptus plantations, 4% others). A more detailed description of the study area can be found in Pardini et al. [16].

### Study sites

This study is part of a larger project investigating the effects of forest fragmentation on the small mammal community in Caucaia do Alto [16,21]. In the overall project several forest fragments differing in size and connectivity to other fragments as well as different plots in the continuous forest of the Morro Grande Forest Reserve were studied. To investigate the microhabitat preferences of small mammals, five forest fragments and one study site in the continuous forest were chosen, so that all sites were of secondary growth forest between 50 and 80 years old [54], but varied widely in terms of fragment size. Two sites were located in fragments of about 14 ha, two in fragments approximately twice as large (30 ha), one in a 175 ha fragment and one within secondary forest inside the Morro Grande Forest Reserve. The study sites were at least 8 km apart and movement of individuals between them was unlikely (and not detected). However, other forest fragments were in the vicinity of the study sites and inter-fragment movement between these fragments was possible.

### Trapping

Regular trapping grids of one hundred trap locations 20 m apart from one another were established in all six study sites. One small and one large trap (23 × 9 × 8 cm and 38 × 11 × 10 cm, respectively; Sherman Traps Inc., Tallahassee, USA) were set up at each trap location. One trap was put on the ground, the other one at an approximate height of 1.0 to 1.5 m, alternating the positions of small and large traps from one trap location to the next.

Data collection was carried out during two trapping sessions in each of the study sites from March to June 2004 (1st session: 4.03.-7.04.2004, 2nd session: 18.05.- 26.06.2004). Each session consisted of six consecutive nights of capture, totalling 2400 trap nights per study site. The traps were baited with banana, and a mixture of peanut butter, oat and sardines. Traps were left open for the night, checked every morning and rebaited if necessary. Captured animals were anaesthetized (Forene^®^, Abbott GmbH, Wiesbaden, Germany) for 1–2 minutes and marked individually by numbered ear tags (Fish and small animal tag size 1, National Band and Tag Co., Newport, Kentucky, USA). In addition to sexing and weighing, the length of their tibia was measured to the nearest 0.5 mm (measurements were taken for another study). All individuals were released at their respective trapping location.

### Vegetation characteristics

Each of the 600 trap locations were characterized with respect to vegetation structure within a 5-meter radius around the trap locations according to August [55]. Vegetation characteristics were canopy height (estimated with the help of a marked 5-meter pole in meters and grouped into seven categories: < 6 m, 6.1–8.0 m, 8.1–10.0 m, 10.1–12.0 m, 12.1–15.0 m, 15.1–20.0 m and 20.1–25 m), canopy cover (estimations were based on a scale from 1 to 4: 1 equals less than 25% cover while 4 counts for 76–100% cover), vegetation density at 0 to 0.5 m, 0.5 to 1.5 m and 1.5 to 3 m (estimations were based on a scale from 1 to 5: 1 indicates 20% of the specific strata is covered by plants, 5 indicates 81–100% of the specific strata is covered by plants), the amount of bamboo (estimations were based on a scale from 1 to 4: 1 represents a percentage of cover of up to 25 of bamboo while 4 represents 76–100 percent bamboo) and the quantity of horizontal structures as an indicator of the connectivity of the vegetation above ground (1–4 scale: 1 means that few or no surrounding trees are connected by horizontal structures, 4 means a 76–100% of the surrounding trees are connected to each other by horizontal structures at any height).

### Statistical analysis

The vegetation variables were standardized and reduced to three principal components (PC1, PC2 and PC3) to minimize correlation and to identify major traits of variation in vegetation structure (SPSS Principal Component Analysis, default settings, Varimax rotation).

We investigated if species distribution is influenced by micro-scale variation in vegetation structure and if these influences depend on the fragment where animals were captured. To test these hypotheses, we compared the scores of the principal components (dependent variables: PC1–PC3) using a factorial ANOVA, with FRAGMENT (1–6) and USE of trap locations (used and unused) as factors. Thus we investigated differences in principal component scores among fragments and between used and unused traps, as well as the existence of interactions between these two factors. Significant interactions indicate that the influence of micro-scale variation in vegetation structure is dependent on the fragment considered.

Only *A. montensis *and *M. incanus *were captured in all fragments. Other species were captured only in five (*D. sublineatus*, *G. microtarsus*) or four (*O. nigripes*) fragments. The component scores of all principal components deviated little from a normal distribution (Kolmogorov-Smirnov-Test). In analysis of variance, non-normality of data can be tolerated, especially when sample size is large [56]. The assumption of homoscedasticity was tested by Levene's test for homogeneity of variances. Variances of PC1 and PC2 differed among fragments. ANOVA is robust to deviation from this assumption where sample size is large [56]. The comparison among fragments included six treatments with n >= 100 each. With many samples or treatments the effect of any one sample variance on the estimated variances within treatments can only be small [56]. Therefore we continued with the analysis although Levene's test indicated heteroscedasticity in PC1 and PC2 among fragments.

We aimed to keep data independent of individual behavioural responses to capture. Therefore, only first-captured individuals of both capture sessions were considered [57]. Trap locations were weighted by the number of individuals captured at the respective location.

All tests were conducted on STATISTICA 6.0 (StatSoft Inc., Tulsa) using a significance level of 0.05.

## Authors' contributions

TP, RP and YML participated in design of the study and data collection. TP performed the statistical analysis. TP and RP wrote the manuscript. SS conceived the study, participated in its design and helped to draft the manuscript. SS and RP participated in the project coordination. All authors read and approved the final manuscript.
